# Integrated backscatter-intravascular ultrasound and modification of plaque during excimer laser coronary angioplasty

**DOI:** 10.1007/s12928-021-00797-0

**Published:** 2021-07-31

**Authors:** Satoru Sasaki, Kenji Nakajima, Keizo Watanabe, Yudai Nozaki, Tadashi Yuguchi, Hiroyuki Sano, Ryo Matsutera, Naoki Murai, Hiroaki Abe, Hideyuki Takaoka

**Affiliations:** 1 Department of Cardiology, Takatsuki General Hospital, Osaka, Japan; 2 Department of Clinical Engineering, Takatsuki General Hospital, Osaka, Japan

**Keywords:** Excimer laser coronary angioplasty, Integrated backscatter-intravascular ultrasound, Plaque modification

## Abstract

This study aims to test the hypothesis that the effect of excimer laser coronary angioplasty (ELCA) not only vaporizes thrombi and their underlying coronary plaque, it also changes their quality. We performed a series of cross-sectional analyses in 52 lesions in 51 patients before and after ELCA with integrated backscatter-intravascular ultrasound (IB-IVUS). The constituent parts of the plaque can be assessed by IB-IVUS (i.e., calcified, fibrous, lipid) according to integrated backscatter values. Minimum lumen diameter, lumen volume and vessel volume expanded after ELCA, while plaque volume did not significantly decrease. There was also a decrease of ‘lipid’ component (35.4–30.3%, *P* < 0.001) and an increase of IB-IVUS-derived ‘fibrous’ part (34.5–38.3%, *P* < 0.001). These results may help in understanding plaque change after ELCA. Excimer laser coronary angioplasty seems to contribute to the modification of coronary plaque composition in addition to debulking it.

**Supplementary Information:**

The online version contains supplementary material available at 10.1007/s12928-021-00797-0.

## Introduction

Excimer laser coronary angioplasty (ELCA) is reported to be useful in percutaneous interventions [1, 2]. This method of atherectomy uses the energy of 308-nm wavelet ultraviolet from excited xenon chloride molecules. The laser breaks the molecular bonds of the plaque tissue and vaporizes the intracellular water, vapor bubbles then break down the radiated tissue [3]. In our clinical experience, we often observed that the media of the target vessel often seems to have higher echogenicity in intravascular ultrasound (IVUS) after ELCA. To our knowledge, however, this phenomenon has not been widely reported. To evaluate the effect of ELCA on the plaque in media of the coronary artery, we used integrated backscatter-intravascular ultrasound (IB-IVUS). We hypothesized that the effect of ELCA might be in part due to debulking the thrombus and its underlying plaque tissue, but also by its modification. We used IB-IVUS to distinguish between different tissue characteristics.

## Methods

### Study design and population

We included patients aged ≥ 20 years with coronary artery disease, and who underwent percutaneous coronary intervention (PCI) in our hospital between August 2018 and March 2020. Patients were included depending on several characteristics. Those with > 90% stenosis on angiogram were included, regardless of symptoms. If patients had > 70% stenosis with symptoms they were also included, as were patients diagnosed with ischemia according to treadmill testing and/or physiological estimation by pressure wire, regardless of the presentation of symptoms. In all cases, the targets were de novo lesions and ELCA was used within the procedures. The lesions were estimated before and after ELCA by IVUS using 60 MHz ultrasound signals (VISICUBE, Terumo, Tokyo, Japan), and all could be visualized by IB-IVUS. Cases were excluded if the patient required cardiopulmonary resuscitation, if the target lesion was restenosis, if PCI was to de novo branch lesions in which the main vessel had been revascularized before, or if balloon angioplasty was performed between the IVUS procedures being compared. The study was approved by the Takatsuki General Hospital Institutional Review Board. The need for individual patient consent was waived because data were collected retrospectively and anonymized. The study flowchart is shown in Supplementary Fig. 1.

### Definition

Diagnosis of ST-segment elevation myocardial infarction (STEMI) was as previously proposed [4]. Diagnosis of unstable angina pectoris/non-ST-segment elevation myocardial infarction (UAP/NSTEMI) requires symptoms consistent with cardiac ischemia and evident stenosis shown by coronary angiography with ST-segment deviations in ECG at rest. It must not meet the STEMI criteria.

Exertional angina pectoris and silent ischemic heart disease are both considered to be stable angina pectoris (AP) and are diagnosed by treadmill exercise test [5], or by physiological examination with pressure wire during coronary angiography [6]. Procedural success was defined as a final reduction of lumen diameter stenosis to < 30% and thrombolysis in myocardial infarction (TIMI) 3 flow in the absence of major in-hospital complications (death, emergency bypass surgery, novel development of myocardial infarction and target vessel revascularization).

### Coronary intervention

We typically pretreated patients with dual antiplatelet therapy: 100 mg of aspirin and a P2Y12 inhibitor of either 75 mg of clopidogrel or 3.75 mg of prasugrel daily for at least five days. If these were not prescribed (e.g., in emergent cases), loading dose of 200 mg of aspirin, 300 mg clopidogrel, or 20 mg prasugrel was administered before the coronary intervention. Intravenous heparin was administered during the index procedure to maintain an activated clotting time > 250 s. Glycoprotein IIa/b inhibitors were not used in any cases.

When angiogram or IVUS revealed thrombus on the target lesion, a thrombus aspiration device was used first. After IVUS analysis, ELCA was then used at the physicians’ discretion.

The pulse-wave xenon chloride excimer laser generator (CVX-300P, Spectranetics, Colorado Spring, Colorado) operates at 308-nm wavelength. Pulse duration is 135 ns and the output is 165 mJ/pulse. Initial energy parameters were set at a fluence of 45 mJ/mm^2^ and 25 Hz, which was increased if the debulking effect was insufficient. The catheter (ELCA, Spectranetics, Colorado Spring, CO) size was chosen from a concentric type of 0.9, 1.4, 1.7, or 2.0 mm, or eccentric type of 1.7 or 2.0 mm in diameter according to the reference of the vessel size and plaque eccentricity. During ablation procedures, saline was injected [7], and the laser catheter was advanced as slowly as 0.5 mm/s [8].

### Quantitative coronary angiography

Angiographic images were analyzed using commercially available software (QAngio XA7.3, Medis Medical Imaging System B.V., Leiden, the Netherlands). We recorded the minimal lumen diameter, reference vessel diameter, percentage of plaque area, and lesion length of each lesion. If lesion length and reference vessel diameter could not be calculated in an occluded vessel, we used the first angiogram in which the lumen was recanalized after the thrombus aspiration.

### IVUS analysis

We administered intracoronary nitroglycerin before the image acquisition at between 100 and 250 μg, depending on the systemic blood pressure. We performed IVUS assessments before and after laser angioplasty. Using the motorized transducer pullback system, the 3.0 Fr IVUS imaging catheter (Altaview, Terumo, Tokyo, Japan) incorporated a 60-MHz phased array transducer (VISICUBE, Terumo, Tokyo, Japan). Offline IVUS images were analyzed by independent investigators who were unaware of patient clinical characteristics. Quantitative IVUS analyses were performed according to the expert consensus document from the Japanese Association of Cardiovascular Intervention and Therapeutics [9]. External elastic membrane, lumen and intima-media complex areas were obtained by manual contour detection. To illustrate the tissue characteristics of the lesion, color-coded maps were constructed for each 0.1–0.5 mm slice. Lumen area, lumen volume, vessel volume, and total plaque volume, and the percentage of each plaque component were automatically calculated. Compositional tissue characteristic areas were expressed as colors according to integrated backscatter values, as previously described (red: calcification, yellow: dense fibrosis, green: fibrosis, blue and purple: lipid pool) [10, 11].

### Statistical analyses

All statistical analyses were performed with EZR (Saitama Medical Center, Jichi Medical University, Saitama, Japan), a graphical user interface for R (The R Foundation for Statistical Computing, Vienna, Austria) [12]. Categorical variables are presented as frequencies with percentage and continuous variables are presented as mean ± standard deviation. Patient data were analyzed before and after ELCA with paired *t* test. Categorical variables were compared using the chi-square or Fisher’s exact test as appropriate. A two-sided *P* value < 0.05 was considered to be statistically significant.

## Results

In line with the current guidelines, PCI for de novo lesions using ELCA and IB-IVUS was performed in our hospital for 72 lesions in 71 patients during the study period. We excluded 20 cases; 14 due to impaired IVUS analysis, four because of lost data and two because of inadequate procedure (balloon angioplasty performed between the index IVUS pullbacks).

### Representative case

**Fig. 1:**
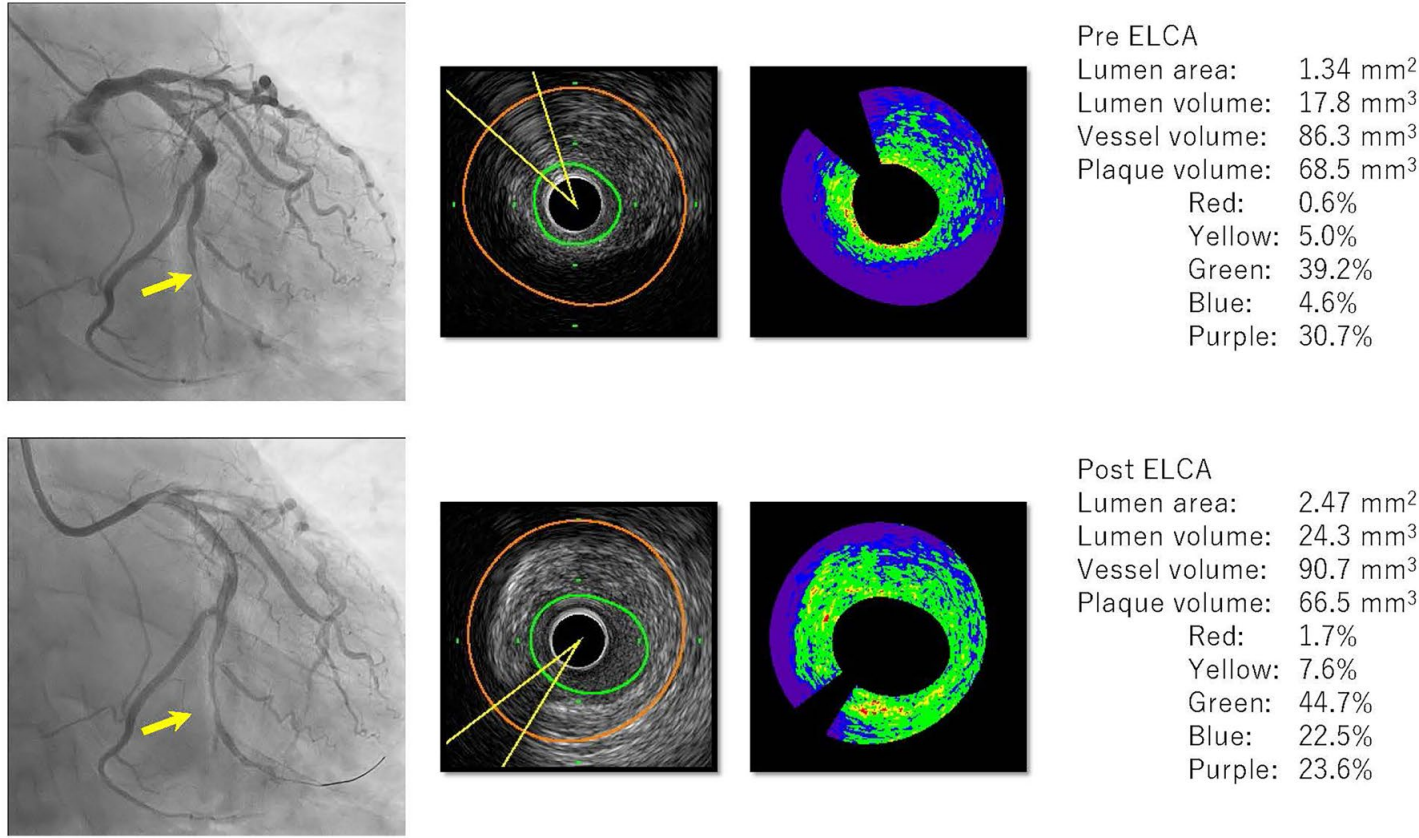
Angiography, conventional IVUS, IB-IVUS images and parameters before and after ELCA of a representative case. Angiography before ELCA (above, left), conventional and IB-IVUS images before ELCA (above middle), IVUS parameters before ELCA (above right). Angiography after ELCA (below, left), conventional and IB-IVUS images after ELCA (below, middle), IVUS parameters after ELCA (below, right). *ELCA* excimer laser coronary angioplasty. *IB-IVUS* integrated backscatter-intravascular ultrasound. *IVUS* intravascular ultrasound

A representative STEMI case is shown in Fig. 1. A 59-year-old male with hypertension and hyperlipidemia was admitted to our hospital with chest pain. Coronary angiography showed severe stenosis with flow limitation in a branch of left circumflex. ELCA was used, followed by balloon angioplasty and stenting of a sirolimus-eluting stent. Conventional IVUS showed that the minimum lumen area (1.34–2.47 mm^2^) and the lumen volume (17.8–24.3 mm^3^) increased during ELCA. IB-IVUS, meanwhile, additionally revealed that plaque composition shifted from being mainly purple-colored (lipid) to being green-colored (IB-IVUS-derived fibrous); purple-colored area, 30.7% to 23.6% green-colored area 39.2% to 44.7%.

### Patient characteristics

Table 1 shows clinical characteristics of the patients in this study. The mean age was 68.3 years, 76% were men, and 25% had diabetes mellitus. STEMI was diagnosed in 50% of patients. Laboratory data showed the average estimated glomerular filtration rate 73 mL/min/1.73 m^2^, low-density lipoprotein cholesterol level 108 mg/dL, hemoglobin A1c 6.3%. On admission, 41% of the patients took aspirin and 37% took P2Y12 receptor inhibitor. All patients who did not take these antiplatelet drugs were administered a loading dose before index PCIs. Echocardiography showed that the average left ventricular ejection fraction of each patient was preserved at 54 ± 11%.

**Table 1:** Patient characteristics

	*n* = 51
Age (years)	68.3 ± 12.3
Male	39 (76%)
BMI	23.6 ± 3.6
Clinical diagnosis	
STEMI	26 (50%)
UAP/NSTEMI	13 (25%)
Stable AP	13 (25%)
Hypertension	33 (65%)
Hyperlipidemia	39 (76%)
Diabetes mellitus	13 (25%)
Smoking	31 (61%)
Family history of CAD	11 (22%)
Chronic kidney disease	12 (24%)
Old myocardial infarction	1 (2%)
Prior PCI	3 (6%)
Prior CABG	0 (0%)
Hemodialysis	1 (2%)
LVEF (%)	54 ± 11
Serum creatinine (mg/dL)	0.97 ± 0.62
Estimated GFR (mL/min/1.73 m^2^)	72.9 ± 28.8
LDL-C (mg/dL)	108 ± 41
HDL-C (mg/dL)	57 ± 18
HbA1c (%)	6.3 ± 1.5
Medication at admission	
Aspirin	21 (41%)
P2Y12 receptor inhibitor	19 (37%)
ACE-I/ARB	18 (35%)
Beta-blocker	7 (14%)
Calcium channel blocker	20 (39%)
Statin	20 (39%)
Insulin	4 (8%)

Data are expressed as the mean ± standard deviation or the number (percentage)

*ACE-I* angiotensin-converting enzyme inhibitor; *AP* angina pectoris; *ARB* angiotensin receptor blocker; *BMI* body mass index; *CABG* coronary artery bypass grafting; *CAD* coronary artery disease; *GFR* glomerular filtration rate; *HbA1c* hemoglobin A1c; *HDL-C* high-density lipoprotein cholesterol; *LDL-C* low-density lipoprotein cholesterol; *LVEF* left ventricular ejection fraction; *OMI* old myocardial infarction; *PCI* percutaneous coronary intervention; *STEMI* ST-segment elevation myocardial infarction; *UAP/NSTEMI* unstable angina pectoris/non-ST-segment elevation myocardial infarction

### Lesion characteristics

Lesion characteristics are shown in Table 2. Target vessels were left anterior descending arteries in 50% of cases. There were 21 cases (40%) without any flow limitation before intervention, and the target vessel was totally occluded in 12 cases (23%). There were 22 cases with evident thrombus localized in the target vessels (i.e., TIMI thrombus scale Grade 2–5) [13].

**Table 2:** Lesion characteristics

	*n* = 52
Lesion location	
LMT	0 (0%)
LAD/Dg	26 (50%)/1 (2%)
LCx	5 (10%)
RCA	20 (38%)
TIMI flow	
3	21 (40%)
2	16 (31%)
1	3 (6%)
0	12 (23%)
TIMI thrombus grade	
0	9 (17%)
1	21 (40%)
2	3 (6%)
3	4 (8%)
4	3 (6%)
5	12 (23%)
ACC/AHA type	
A	10 (19%)
B1	10 (19%)
B2	20 (38%)
C	10 (19%)
Non-measurable	2 (4%)

Data are expressed as the number (percentage)

*ACC/AHA* American College of Cardiology/American Heart Association; *Dg* diagonal branch; *LAD* left anterior descending artery; *LCx* left circumflex artery; *LMT* left main trunk; *RCA* right coronary artery; *TIMI* thrombolysis in myocardial infarction

### Procedure and angiographic outcomes

The procedure and its outcomes are depicted in Table 3. Relatively smaller catheter sizes (1.4 mm, 0.9 mm) were used in 33 cases (63%). Stent implantation was performed in 32 cases (62%), while all cases used adjunctive balloon angioplasty. Final TIMI 3 flow was achieved in 51 cases (98%), and the procedure was successful in 43 cases (83%). Cases of procedural failure consist of one (typical) case with remaining coronary dissection at the distal edge of the stent, and seven (atypical) cases of paclitaxel drug-coated balloon alone angioplasty with remaining intermediate stenosis. In these cases, stent-free strategy was preferred by PCI operators because of the clinical backgrounds. Clinically, there was no target vessel revascularization and no major adverse cardiac events in any of the seven cases after the index PCI during 234 to 596 days observational periods (data not shown).

**Table 3:** Procedure and angiographic outcomes

		*n* = 52
Laser catheter size		
1.7 mm	19 (37%)	
1.4 mm	23 (44%)	
0.9 mm	10 (19%)	
Laser passes	2 ± 1 (1–5)	
Laser output (mJ/mm^2^)	45–60	
Laser pulses delivered	1522 ± 1022 (375–5395)	
Adjunctive balloon angioplasty	52 (100%)	
Stent implantation	32 (62%)	
Final TIMI 3 flow	51 (98%)	
Procedural success	43 (83%)	

Data are expressed as the number (percentage), the mean ± standard deviation (minimum to maximum)

*TIMI* thrombolysis in myocardial infarction

ELCA followed by angioplasty or stent implantation resulted in the increased minimum lumen (stent) diameter in this study (Table 4). According to quantitative coronary angiogram analysis, minimum lumen diameter expanded (0.81–2.33 mm), and percentage of diameter stenosis decreased (69.7–18.6%).

**Table 4:** Quantitative angiographic results before and after ELCA

	Pre-ELCA (*n* = 50)	Post-ELCA (*n* = 20)	Final (*n* = 52)
MLD (mm)	0.81 ± 0.40	1.21 ± 0.48	2.33 ± 0.48
Reference (mm)	2.65 ± 0.62	2.57 ± 0.63	2.88 ± 0.40
% DS (%)	69.7 ± 12.3	51.8 ± 17.6	18.6 ± 12.5
Lesion length (mm)	14.4 ± 8.3		
Acute gain (mm)		0.33 ± 0.45	1.54 ± 0.58

Data are expressed as the mean ± standard error

*ELCA* excimer laser coronary angioplasty; *MLD* minimum lumen diameter; *%DS* percentage of diameter stenosis

### IVUS analysis

**Fig. 2:**
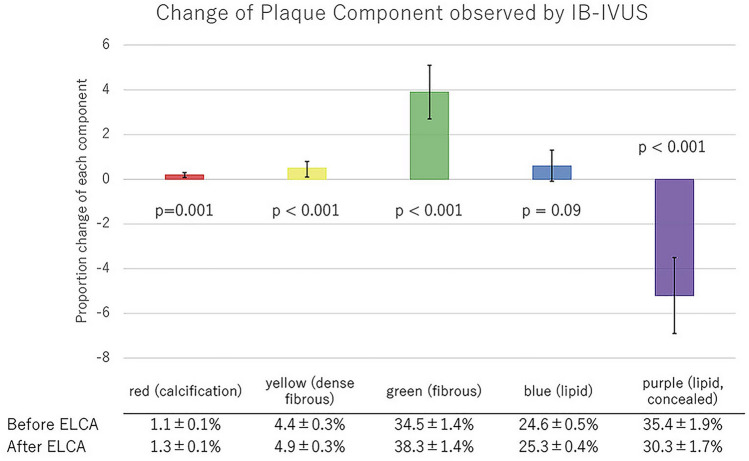
Change of plaque component observed by IB-IVUS

**Fig. 3:**
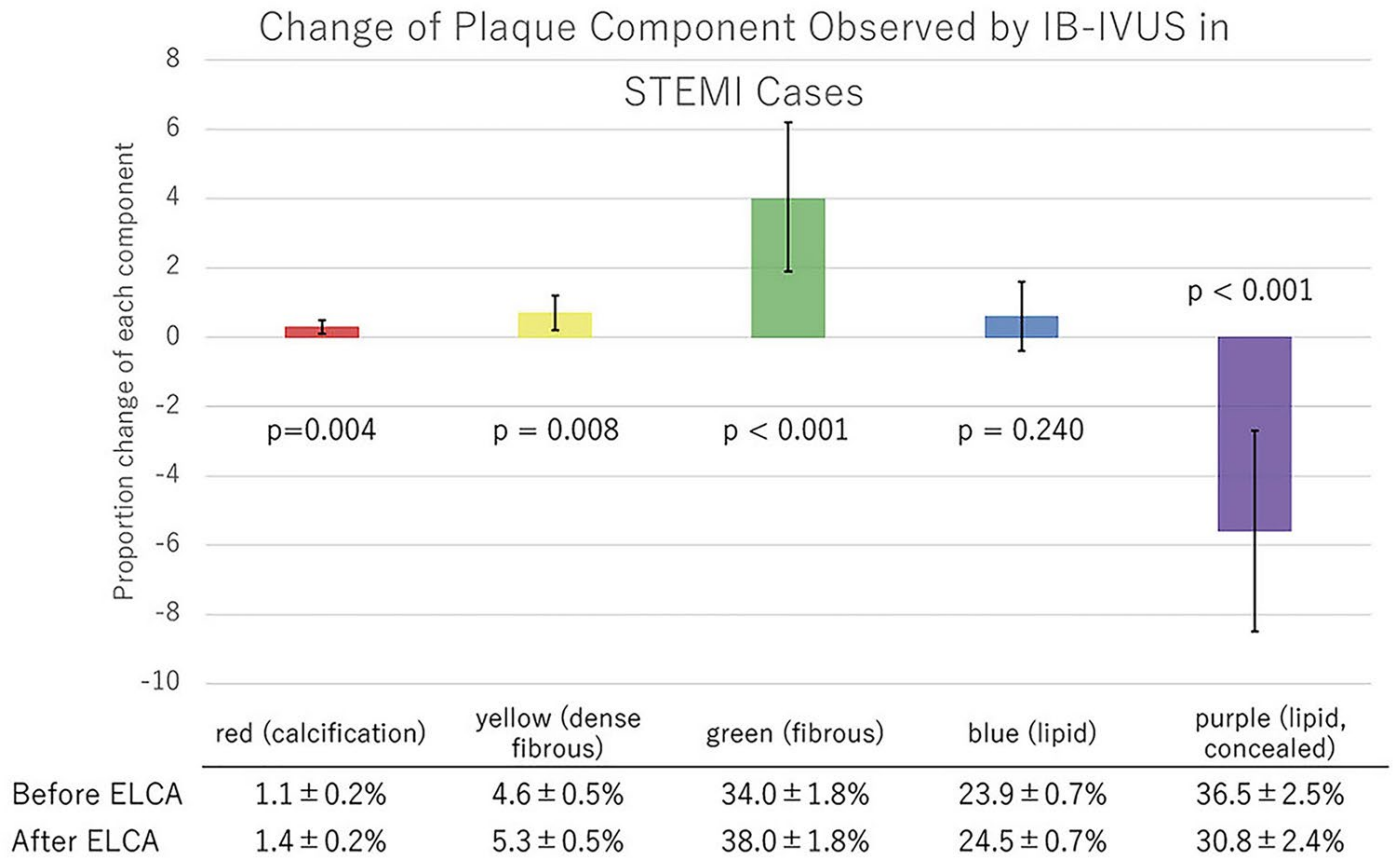
Change of plaque component observed by IB-IVUS in STEMI cases

**Fig. 4:**
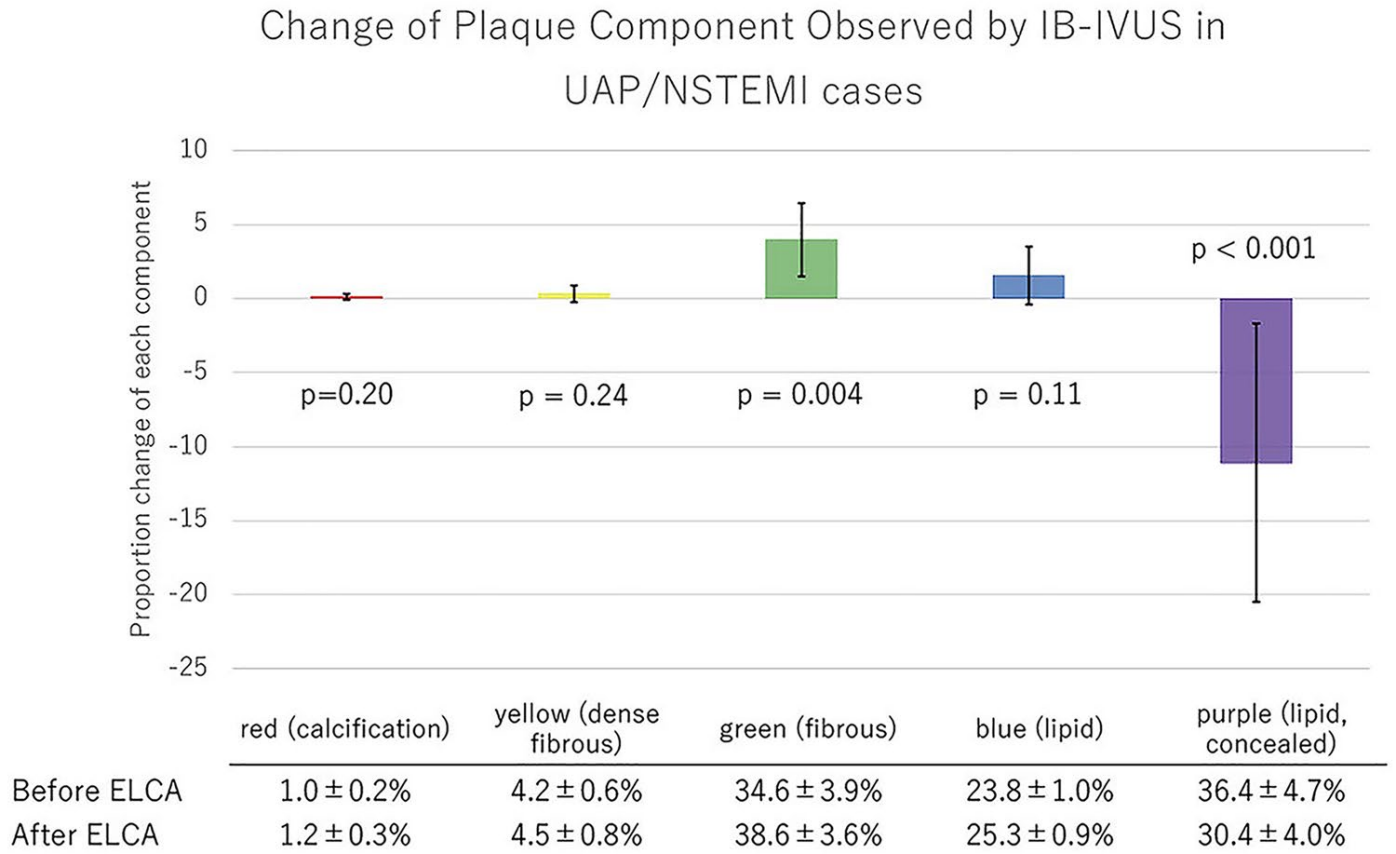
Change of plaque component observed by IB-IVUS in UAP/NSTEMI cases

**Fig. 5:**
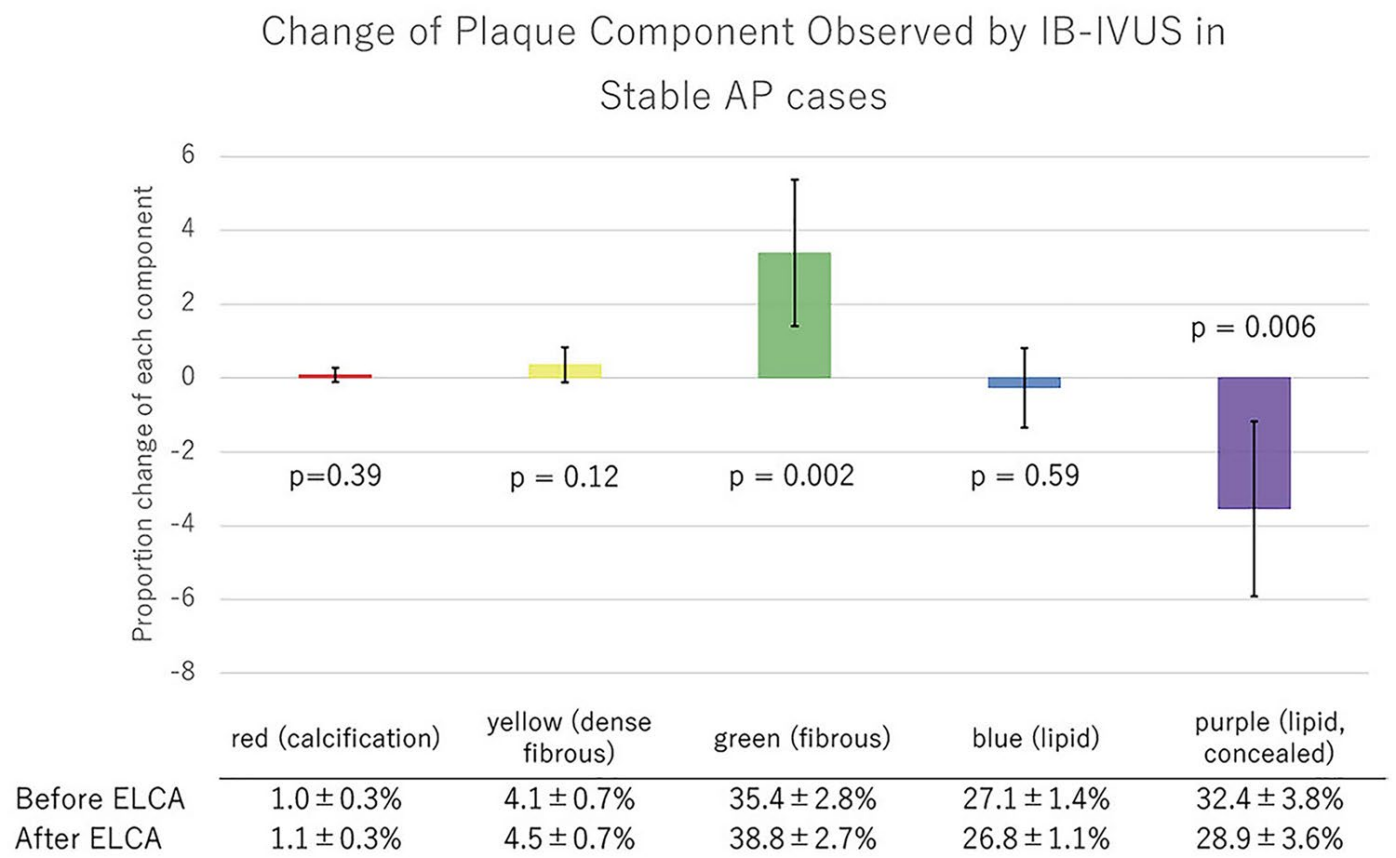
Change of plaque component observed by IB-IVUS in stable AP cases

Quantitative parameters of conventional IVUS are shown in Table 5. Minimum lumen area, lumen volume, and the vessel volume increased significantly after ELCA, while plaque volume did not change. According to subgroup analysis, lumen volume increased in STEMI groups, but did not in the stable AP group. Plaque volume, meanwhile, did not change significantly in any subgroups (Table 5). Assessment of IB-IVUS revealed a decrease in the ratio of purple-colored area (lipid plaque, concealed component), while the green area (IB-IVUS-derived fibrous plaque) increased significantly (Fig. 2). This phenomenon was consistent across all subgroups (Figs. 3, 4, 5). The change in volume of each component corresponded with its ratio. (Supplementary Fig. 2-1, 2-2a, 2b, 2c).

**Table 5:** Quantitative parameters of conventional IVUS

	Pre-ELCA	Post-ELCA	*P*
All cases			
MLA (mm^2^)	1.65 ± 0.07	2.25 ± 0.14	< 0.001
Lumen volume (mm^3^)	43.7 ± 4.0	54 ± 4.7	< 0.001
Vessel volume (mm^3^)	200 ± 19	207 ± 19	0.001
Plaque volume (mm^3^)	156 ± 16	153 ± 15	0.09
STEMI cases			
MLA (mm^2^)	1.64 ± 0.13	2.16 ± 0.15	< 0.001
Lumen volume (mm^3^)	40.8 ± 4.2	50.1 ± 4.7	< 0.001
Vessel volume (mm^3^)	193.7 ± 21.2	202.4 ± 22.5	0.001
Plaque volume (mm^3^)	152.9 ± 19.0	152.3 ± 19.1	0.83
UAP/NSTEMI cases			
MLA (mm^2^)	1.58 ± 0.13	2.23 ± 0.21	0.02
Lumen volume (mm^3^)	46.8 ± 9.4	63.5 ± 13.6	0.05
Vessel volume (mm^3^)	216 ± 47.3	227.3 ± 50.4	0.04
Plaque volume (mm^3^)	169.2 ± 40.0	163.8 ± 37.1	0.21
Stable AP cases			
MLA (mm^2^)	1.75 ± 0.14	2.45 ± 0.43	0.08
Lumen volume (mm^3^)	46.4 ± 10.4	52.1 ± 9.7	0.10
Vessel volume (mm^3^)	195.4 ± 43.1	194.8 ± 40.9	0.90
Plaque volume (mm^3^)	146.7 ± 33.2	142.4 ± 31.8	0.06

Data are expressed as the mean ± standard error

*ELCA* excimer laser coronary angioplasty; *MLA* minimum lumen area; *STEMI* ST-segment elevation myocardial infarction; *UAP/NSTEMI* unstable angina pectoris/non-ST-segment elevation myocardial infarction

## Discussion

This is the first report in which quantitative or qualitative changes in coronary plaque have been estimated by IB-IVUS during ELCA, and it presents two major findings. First, after ELCA, the vessel volume and lumen volume increased, confirming the results of a previous study in 1995 [14]. Lumen enlargement was reported to be gained by tissue ablation and/or vessel expansion without calcium ablation, but there were no predictors of which factor contributed to the lumen enlargement in angiographic or IVUS findings.

In our study, plaque volume was not decreased with significant difference. Lumen enlargement and vessel expansion without calcium ablation were found mainly in cases of STEMI, and we suggest the same could be said of focal tissue ablation.

The reason for enlargement of the lumen and the vessel being observed in the STEMI group but not in the stable AP group is unclear. Vessel compliance and plaque morphology may be different between these two groups. The difference in morphological pattern between the acute coronary syndrome group and the stable AP group is shown in Figs. 2, 3, 4, and 5.

A second finding was that there was a decrease of purple-colored areas (lipid tissue) in color maps of IB-IVUS, but an increase of green-colored areas (fibrous tissue) after ELCA. This is interesting phenomenon because there was no statistically significant decrease in plaque volume. From a biochemical point of view, it is difficult to believe that lipid-rich plaque changed into fibrous plaque, it is easier to assume that lipid is modified with high echogenicity. Such modification was not shown, however, in a previous report of ex vivo histological assessment in microscopy, in which excimer laser did not injure aortic media postmortem [15]. This modification of lipid plaque has thus not yet been histologically identified. How the effect of excimer laser results in this alteration is unclear. Although harmful damage has not been reported, change of up to approximately 10 °C has been observed in vitro assays [16], so the change may have arisen from thermal effect.

We believe that the effect of ELCA was solely dependent upon the increase of lumen volume by plaque debulking. Lumen enlargement by vessel volume expansion and morphological change of intima–media complex in coronary arteries were implied by our study to be other effects of ELCA.

PCI operators sometimes encounter cases in which the lumen is not satisfactorily enlarged after ELCA. There is hesitation to increase the energy setting of ELCA owing to the potential for coronary perforation. We may conclude that ELCA reaches its optimal result when the green-colored area by IB-IVUS increased after ELCA. Clarification of the feasibility of this conservative method by comparison of this conservative ablation method with aggressive ablation (gaining lumen volume) methods will be the subject of further studies.

### Limitations

This study has some limitations; it is a single-center retrospective study with a small patient population, and it is not a controlled trial, so the results observed by IB-IVUS cannot be gained just by ELCA. There were four cases in which IB-IVUS examination was performed before and after angioplasty prior to ELCA, implying that the purple-colored area decreased only balloon angioplasty (Supplementary table). Further study is, therefore, essential for comparison between the ELCA group and non-ELCA group. Although ELCA was expected to be used to debulk thrombi and plaque, there was no significant decrease in plaque volume in this study. This means that ELCA may be insufficient in this study. It is, however, interesting that the morphologic pattern of IB-IVUS shifted despite the insufficient debulking effect in volume.

Distinguishing thrombi from medial plaque is also difficult, especially in STEMI cases. Thrombi tend to be categorized as blue- or purple-colored components (lipid tissue) and can hide underlying plaque, which may result in underestimation of other categories. Future studies should aim to pathologically solve this intrinsic limitation of IVUS.

## Conclusion

Excimer laser coronary angioplasty seems to contribute to the modification of coronary plaque composition in addition to debulking it. These results may illuminate understanding about plaque change after ELCA.

## Supplementary Information

Below is the link to the electronic supplementary material.

Supplementary file1 (JPG 123 KB)

Supplementary file2 (JPG 169 KB)

Supplementary file3 (JPG 168 KB)

Supplementary file4 (JPG 174 KB)

Supplementary file5 (JPG 167 KB)

Supplementary file6 (JPG 94 KB)

## Abbreviations

AP: Angina pectoris
ELCA: Excimer laser coronary angioplasty
IB-IVUS: Integrated backscatter-intravascular ultrasound
PCI: Percutaneous coronary intervention
STEMI: ST-segment elevation myocardial infarction
TIMI: Thrombolysis in myocardial infarction
UAP/NSTEMI: Unstable angina pectoris/non-ST-segment elevation myocardial infarction

## References

[1] Topaz O, Ebersole D, Das T, Alderman EL, Madyoon H, Vora K (2004). Excimer laser angioplasty in acute myocardial infarction (the CARMEL multicenter trial). Am J Cardiol.

[2] Nishino M, Mori N, Takiuchi S, Shishikura D, Doi N, Kataoka T (2017). Indications and outcomes of excimer laser coronary atherectomy: efficacy and safety for thrombotic lesions—the ULTRAMAN registry. J Cardiol..

[3] Egred M, Brilakis ES (2020). Excimer laser coronary angioplasty (ELCA): fundamentals, mechanism of action, and clinical applications. J Invasive Cardiol.

[4] Thygesen K, Alpert JS, Jaffe AS, Simoons ML, Chaitman BR, White HD (2012). Joint ESC/ACCF/AHA/WHF Task Force for the Universal Definition of Myocardial Infarction. Third universal definition of myocardial infarction. Circulation.

[5] Fletcher GF, Ades PA, Kligfield P, Arena R, Balady GJ, Bittner VA (2013). American Heart Association Exercise, Cardiac Rehabilitation, and Prevention Committee of the Council on Clinical Cardiology, Council on Nutrition, Physical Activity and Metabolism, Council on Cardiovascular and Stroke Nursing, and Council on Epidemiology and Prevention. Exercise standards for testing and training: a scientific statement from the American Heart Association. Circulation..

[6] Neumann F-J, Sousa-Uva M, Ahlsson A, Alfonso F, Banning AP, Benedetto U (2018). ESC/EACTS Guidelines on myocardial revascularization. EuroIntervention.

[7] Deckelbaum LI, Natarajan MK, Bittl JA, Rohlfs K, Scott J, Chisholm R (1995). The Percutaneous Excimer Laser Coronary Angioplasty (PELCA) Investigators. Effect of intracoronary saline infusion on dissection during excimer laser coronary angioplasty: a randomized trial. J Am Coll Cardiol.

[8] Topaz O (1993). A new, safer lasing technique for laser-facilitated coronary angioplasty. J Interv Cardiol.

[9] Saito Y, Kobayashi Y, Fujii K, Sonoda S, Tsujita K, Hibi K (2020). Clinical expert consensus document on standards for measurements and assessment of intravascular ultrasound from the Japanese Association of Cardiovascular Intervention and Therapeutics. Cardiovasc Interv Ther.

[10] Kawasaki K (2015). An integrated backscatter ultrasound technique for the detection of coronary and carotid atherosclerotic lesions. Sensors (Basel).

[11] Okubo M, Kawasaki M, Ishihara Y, Takeyama U, Yasuda S, Kubota T (2008). Tissue characterization of coronary plaques: comparison of integrated backscatter intravascular ultrasound with virtual histology intravascular ultrasound. Circ J.

[12] Kanda Y (2013). Investigation of the freely available easy-to-use software 'EZR' for medical statistics. Bone Marrow Transplant.

[13] Gibson CM, de Lemos JA, Murphy SA, Marble SJ, McCabe CH, Cannon CP (2001). TIMI Study Group. Combination therapy with abciximab reduces angiographically evident thrombus in acute myocardial infarction: a TIMI 14 substudy. Circulation..

[14] Mintz GS, Kovach JA, Javier SP, Pichard AD, Kent KM, Popma JJ (1995). Mechanisms of lumen enlargement after excimer laser coronary angioplasty. An intravascular ultrasound study. Circulation.

[15] Grundfest WS, Litvack F, Forrester JS, Goldenberg T, Swan HJ, Morgenstern L (1985). Laser ablation of human atherosclerotic plaque without adjacent tissue injury. J Am Coll Cardiol.

[16] Papaioannou T, Yadegar D, Vari S, Shehada R, Grundfest WS (2001). Excimer laser (308 nm) recanalisation of in-stent restenosis: thermal considerations. Lasers Med Sci.

