# Supplementary figures and images for: Integrated backscatter-intravascular ultrasound and modification of plaque during excimer laser coronary angioplasty
Source: Cardiovasc Interv Ther. 2021 Jul 31;37(2):354–62. doi: 10.1007/s12928-021-00797-0 (PMC8926960; doi:10.1007/s12928-021-00797-0)

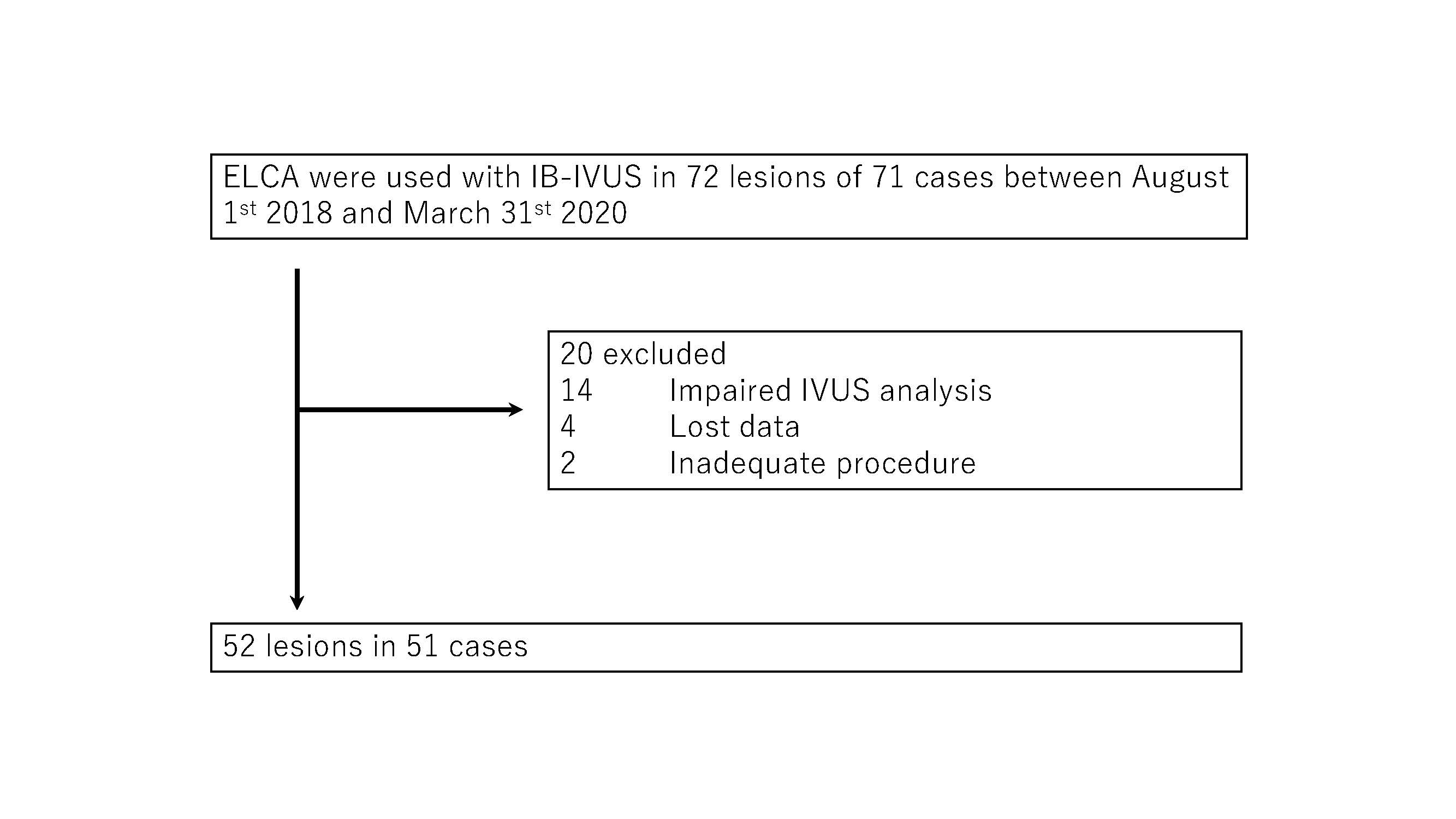

Supplement: Supplementary file 1 — Supplementary file1 (JPG 123 KB) [file 12928_2021_797_MOESM1_ESM.jpg]

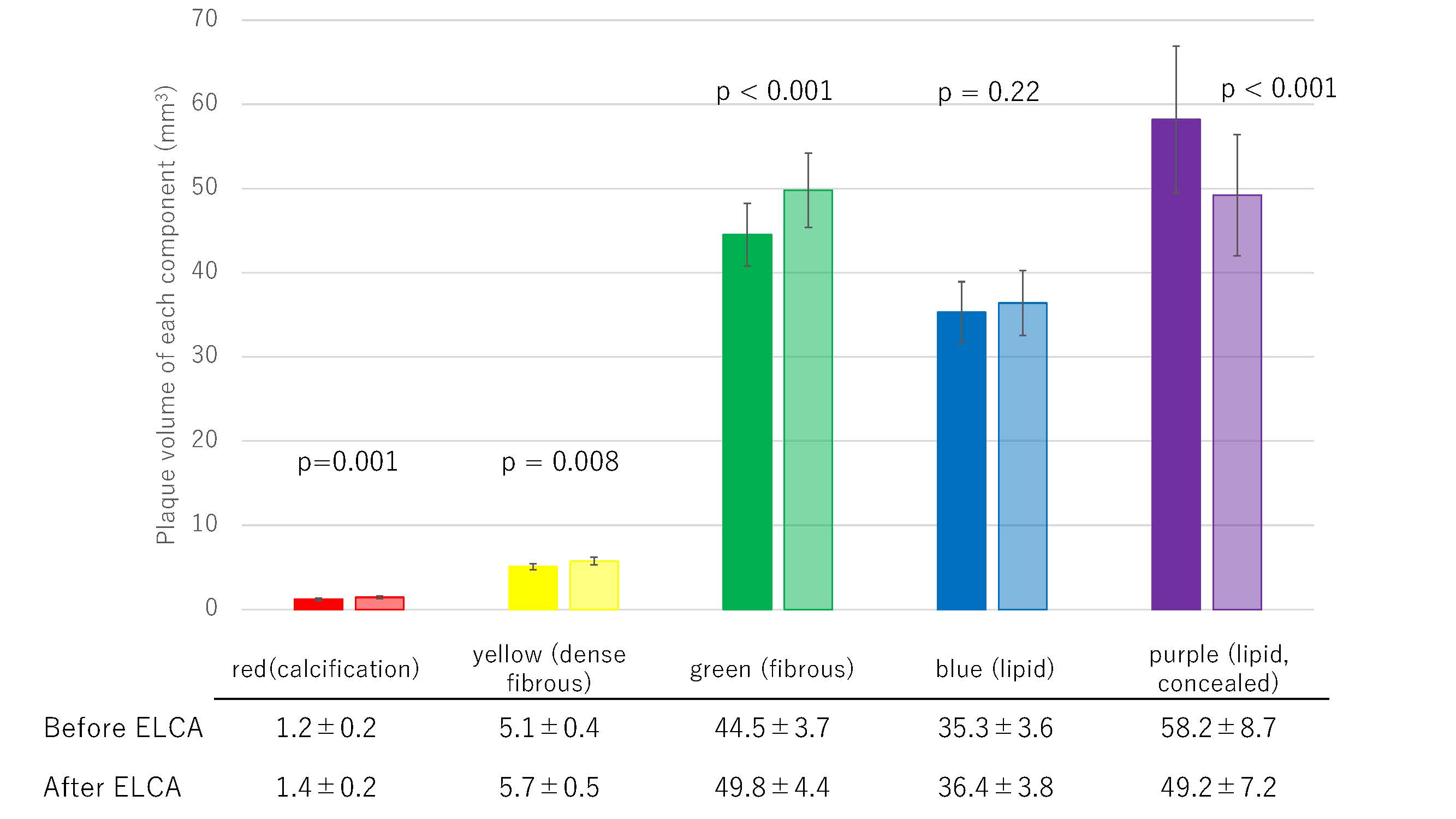

Supplement: Supplementary file 2 — Supplementary file2 (JPG 169 KB) [file 12928_2021_797_MOESM2_ESM.jpg]

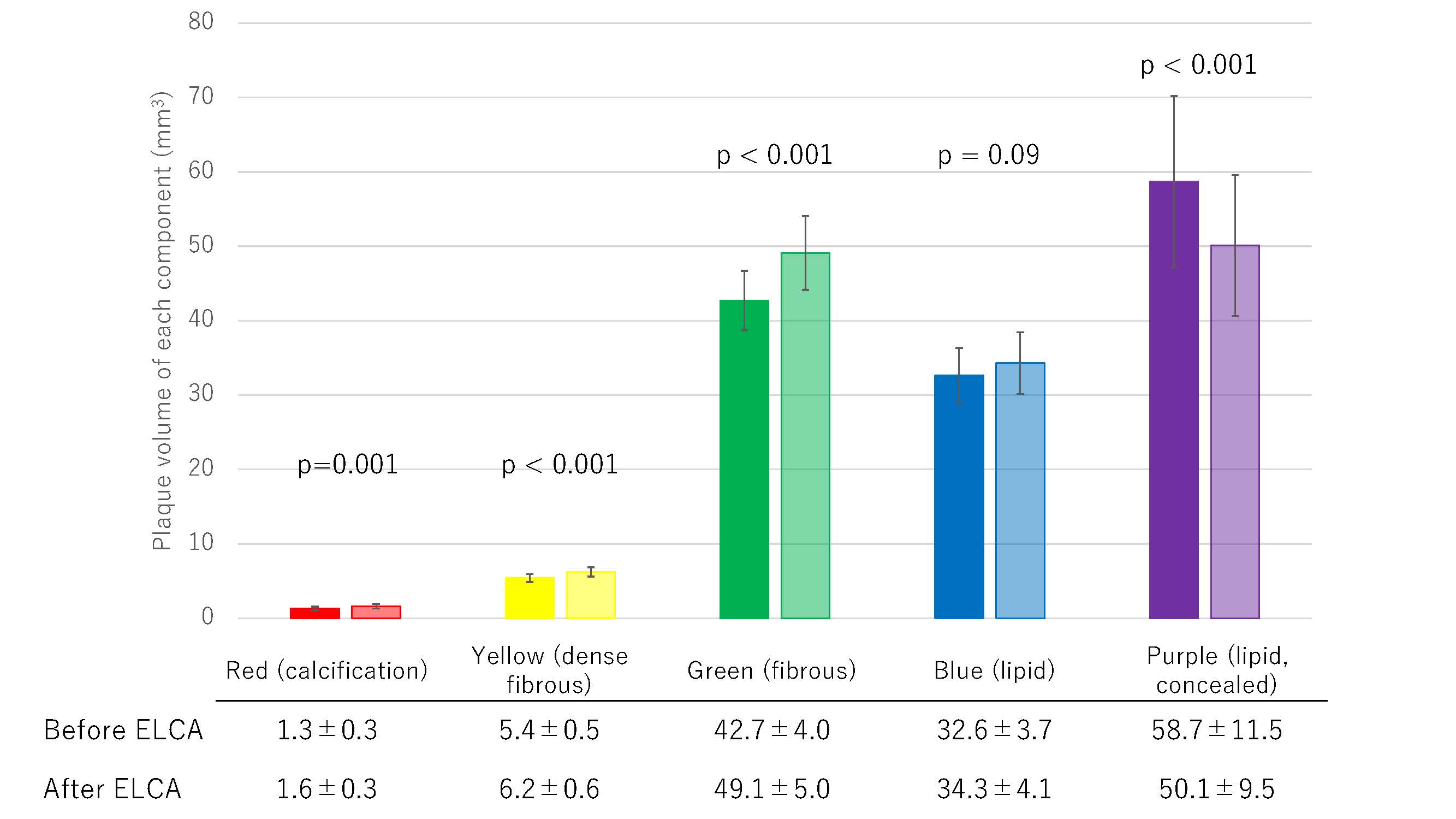

Supplement: Supplementary file 3 — Supplementary file3 (JPG 168 KB) [file 12928_2021_797_MOESM3_ESM.jpg]

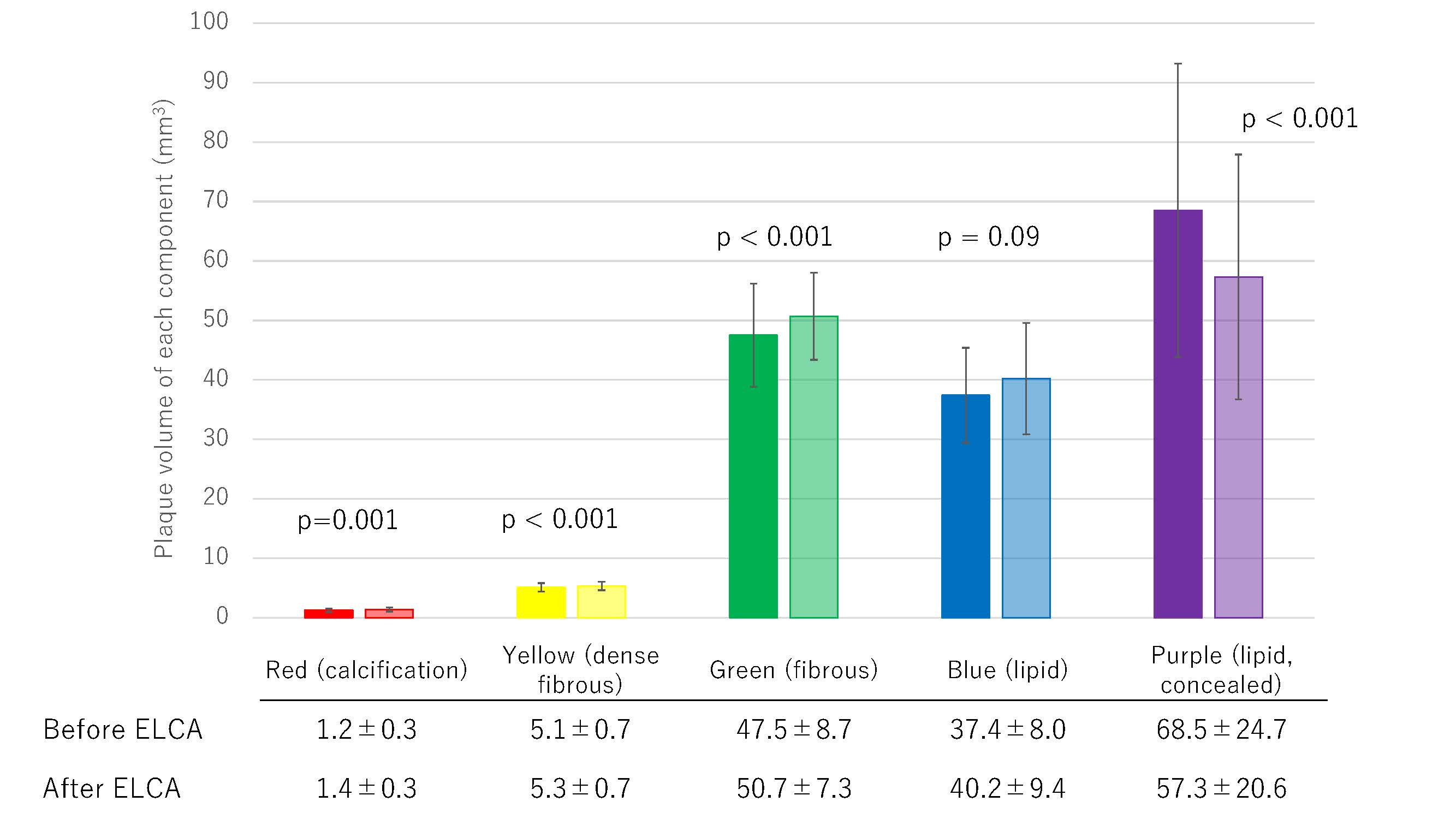

Supplement: Supplementary file 4 — Supplementary file4 (JPG 174 KB) [file 12928_2021_797_MOESM4_ESM.jpg]

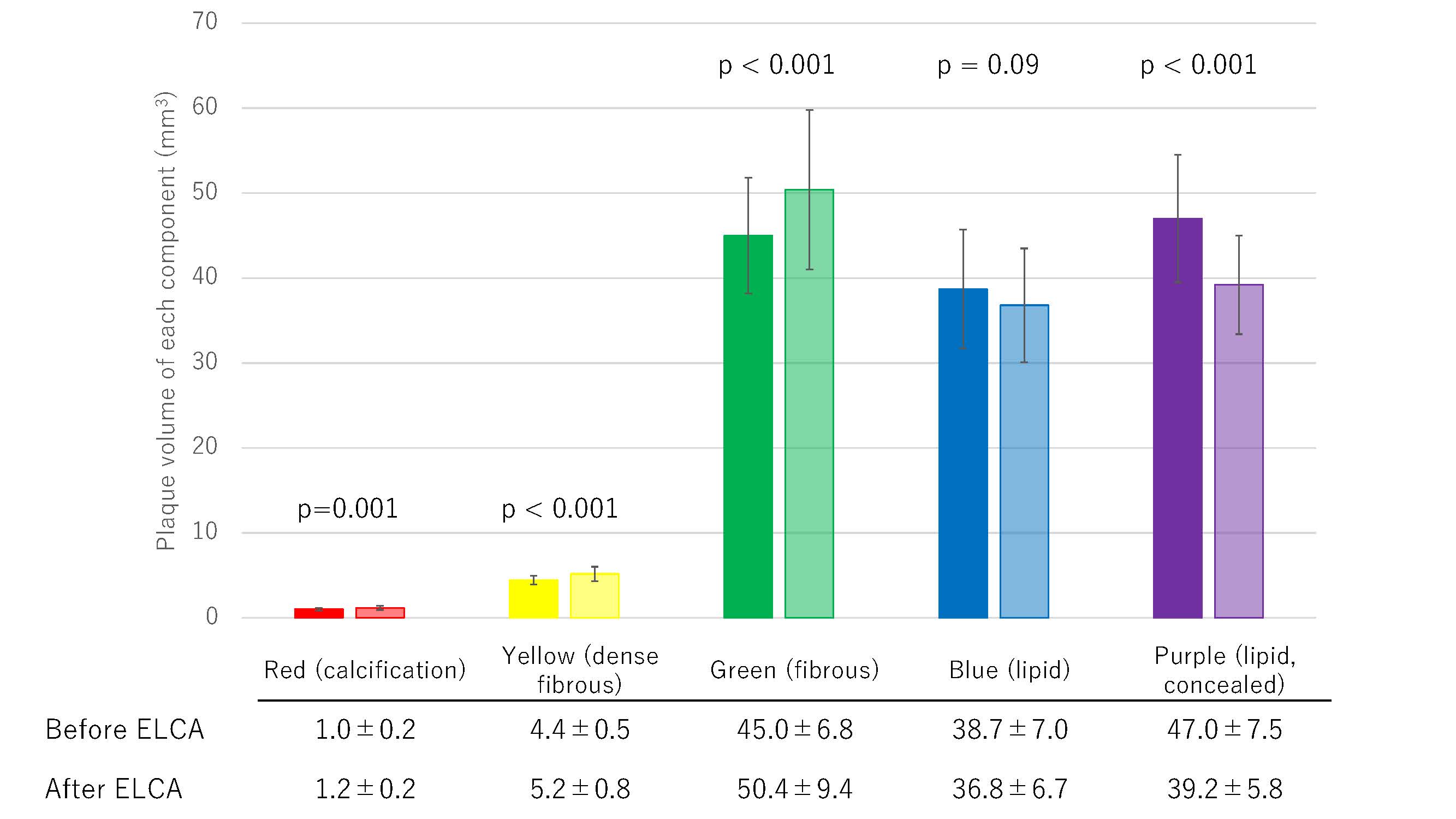

Supplement: Supplementary file 5 — Supplementary file5 (JPG 167 KB) [file 12928_2021_797_MOESM5_ESM.jpg]

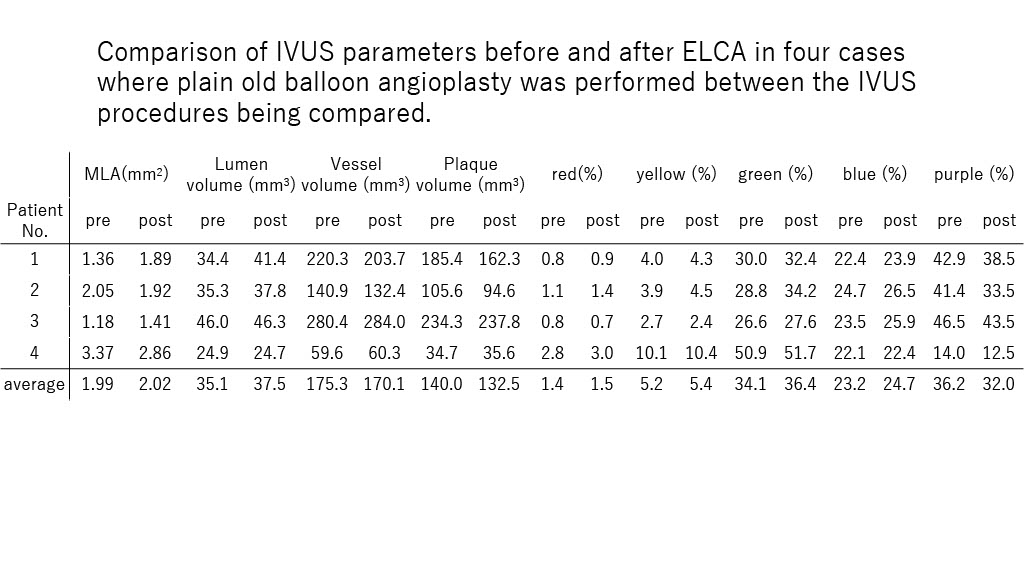

Supplement: Supplementary file 6 — Supplementary file6 (JPG 94 KB) [file 12928_2021_797_MOESM6_ESM.jpg]
